# Crossed renal ectopia with fusion in a pelvic inlet area, atypical portal vein and coccygeal deformation in a young female cat

**DOI:** 10.1186/s12917-020-02535-9

**Published:** 2020-08-27

**Authors:** Mateusz Hebel, Jakub Jędrzej Ruszkowski, Elżbieta Giza, Małgorzata Pomorska-Mól

**Affiliations:** 1 Department of Internal Medicine and Diagnostics, Faculty of Veterinary Medicine and Animal Sciences, Poznan University of Life Sciences, Wolynska 35, 60-637 Poznan, Poland; 2 Department of Animal Anatomy, Faculty of Veterinary Medicine and Animals Sciences, Poznan University of Life Sciences, Wojska Polskiego 71C, 60-625 Poznan, Poland; 3 Department of Preclinical Sciences and Infectious Diseases, Faculty of Veterinary Medicine and Animal Sciences, Poznan University of Life Sciences, Wolynska, 35 Poznań, Poland

**Keywords:** Cat, Congenital defect, Renal ectopia, CT

## Abstract

**Background:**

The case report describes a rare congenital anomaly, crossed fused renal ectopia (CFRE), with coexistence of two other abnormalities – atypical portal vein and coccygeal vertebrae malformation in a domestic cat. The concomitance of those 3 congenital defects has not been described previously.

**Case presentation:**

An 8-month-old female, domestic cat suffering from chronic diarrhea was referred to the diagnostic imaging unit. The patient showed no other clinical symptoms. An abdominal ultrasonographic examination was performed in order to evaluate the condition of abdominal organs, particularly the gastrointestinal tract. The ultrasound examination showed an ectopic duplex kidney at instead of kidneys in their typical location. Computed tomography (CT) with angiographic phase and excretory urography was requested to evaluate the condition of the kidneys and ureters. The final diagnosis was CFRE, atypical portal vein and coccygeal deformation in an asymptomatic cat with no changes in renal function and normal blood parameters.

**Conclusions:**

Crossed fused renal ectopia is a rare congenital anomaly and is easily detectable by an abdominal ultrasonographic examination and CT, which allows more complete assessment of both anatomical relations and secretory function of the kidney. The occurrence of CFRE, abnormal portal vein and spinal malformation in a clinically healthy patient is the evidence that congenital malformations may simultaneously involve various, not directly related structures and systems, without significant influence on blood and urine parameters. Thus the most useful tool in the evaluation of the morphological and functional changes is the diagnostic imaging, especially contrast enhanced CT. Our results show that renal fusions should be considered in the differential diagnosis of caudal abdominal masses.

## Background

Urinary system development is a complicated process of interactions between the embryonic precursors of kidneys, ureters, bladder, and urethra [1]. Congenital malformations of the urinary system, especially the kidneys, are not the problem commonly described in veterinary medicine, and they are rarely the cause of clinical problems [2, 3]. Many authors refer to data from human medicine literature, where kidney malformations are described in more detail than in companion animals such as dogs and cats [2–5]. Renal ectopia, including crossed fused renal ectopia (CFRE) and horseshoe kidney, can be found among these kidney malformations.

Ectopic kidneys have previously been described as a defect in humans [6], cattle [7], dogs [2, 8] and cats [3–5, 9, 10], most often as asymptomatic and appearing rarely. Until now, CFRE cases in cats were reported in both, males [3, 9] and females [4, 5, 10]. Developmental defects, especially CFRE, may predispose to the occurrence of nephrolithiasis, hydronephrosis or urinary tract infections resulting in pyelonephritis [3].

To date, five reports of CFRE in cats have been described [3–5, 9, 10]. Modern methods of diagnostic imaging allow for better detection of this type of congenital defects in vivo, and reports describing the condition can be extremely helpful in the differential diagnosis of the masses in the caudal part of the abdominal cavity [3].

## Case presentation

An eight-month, female domestic cat was referred for abdominal ultrasonographic (USG) examination due to recurrent diarrhoea. The previous medical history of patient was unknown. Medical records provided by the owner lack the information on vaccinations and antiparasitic prophylaxis. During the visit, the condition of the cat was normal: body condition score (BCS) 3/5, pink mucous membranes, capillary refill time (CRT) < 2 s, and 38.5 °C of rectal temperature. Chest auscultation did not reveal any abnormalities in the lungs or heart. Femoral pulse measured at femoral artery was 140 beats per minute (bpm), symmetrical, steady, even and full. Systolic blood pressure measured by the doppler method on the palmar surface of the left thoracic limb was 140 mmHg. The palpation of the caudal abdomen revealed no kidneys in the lumbar region. However, in the caudal abdominal area, however, a solid, painless and non-shifting mass was found. The cat was negative for feline immunodeficiency virus (FIV) antibody and feline leukaemia virus (FeLV) (FIV/FeLV Test SNAP, Idexx, Germany).

**Fig. 1:**
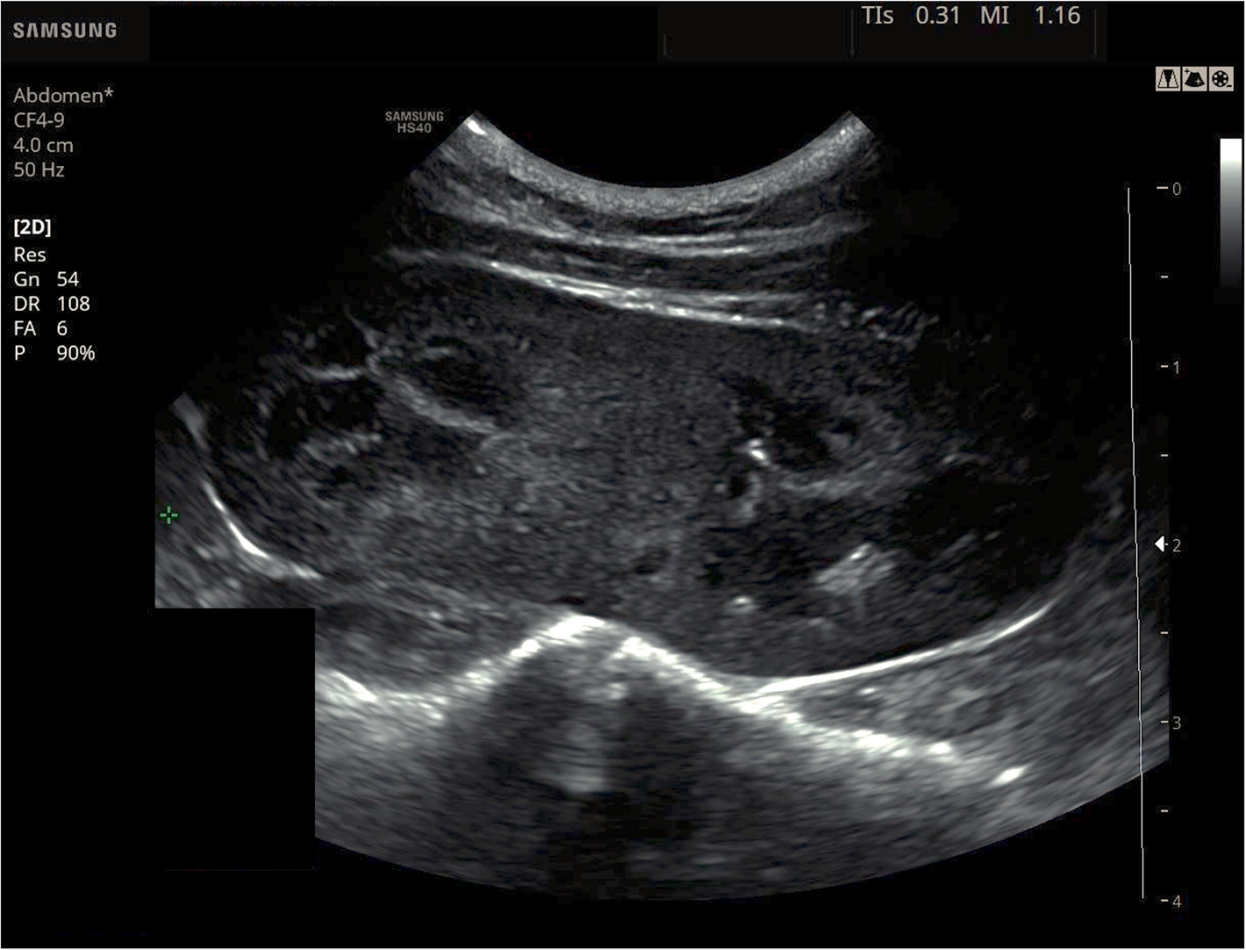
Abdominal sonogram in sagittal plane showing a duplex kidney with fusion at the poles. The renal corticomedullary distinction is preserved and the echogenicity of the renal parenchyma normal

Ultrasonographic examination of the abdomen was performed using an ultrasound scanner (HS40, Samsung, Seongnam, Republic of Korea) with a 50 Hz, microconvex transducer CF4–9 (Fig. 1). The patient was withheld from food for 8 h before examination, the ventral hair was shaved and acoustic gel was applied on the examined area. The patient was positioned in dorsal recumbency. Ultrasonographic examination confirmed no kidneys in their anatomical position. Instead, an ectopic duplex kidney with dimensions of about 50 mm × 25 mm, located in the caudal abdomen, at the pelvic inlet area, between the spine and the rectum was revealed. There were no abnormalities in the underlying kidney structure, corticomedullary differentiation of the kidney was preserved, and the corticomedullary ratio was 1:1 (Fig. 1). The kidney vascularization in the colour doppler test was normal, the vascular resistance index (RI) measured in the arcuate arteries at the renal poles were within the reference range, RI = 0.71. Two renal pelvises were found, however, due to the location of the kidney, it was not possible to assess the ureters and their orifices in a standard USG examination. The ultrasound image of the remaining abdominal organs was normal. The patient was referred for contrast enhanced computed tomography (CT) of the abdominal and pelvic cavities to assess the kidney perfusion, secretory function and ureter morphology.

Blood morphology, biochemistry tests, and a ventrodorsal radiograph (X-ray) of the abdominal cavity proceded the computed tomography examination. The X-ray was performed using a Gierth HF 200 lamp (Gierth X-Ray International GbmH, Germany) with 60 kVp and 8mAs and a Vivix-s 1417 N (Anyang, Republic of Korea) digital direct radiography detector. The results of hematological and biochemical parameters are summarized in Table 1. Urine and blood tests showed no abnormalities. Urine specific gravity was 1.020 mOsm / kg (reference range 1.016–1.064).

**Table 1:** Hematological (Horiba ABX, Horiba Medical, Japan) and biochemical (Mindray BS-120, Mindray Medical International Co., Ltd., China) parameters of the patient

Parameter	Result	Reference values [11, 12]
**Hematology**		
White blood cell (× 10^3^/mm^3^)	8.5	5.5–19.5
Red blood cell (× 10^6^/mm^3^)	7.39	5.8–10.7
Hemoglobin **(g/dL)**	12	9–15
Hematocrit (%)	34.7	30–47
Platelet (×10^3^/mm^3^)	324	300–800
Mean corpuscular volume (mm^3^)	47	41–51
Mean corpuscular hemoglobin (pg)	16.2	13–18
Mean corpuscular hemoglobin concentration (g/dL)	34.6	31–35
Red blood cell distribution width (%)	17.3	17–22
Mean platelet volume (mm^3^)	10.6	6.5–15
**Electrolytes**		
Sodium (**mmol/L)**	150	143–155
Potassium (**mmol/L)**	4.5	4.1–5.6
Chlorides (**mmol/L)**	115	102–118
**Biochemistry**		
Alanine aminotransferase **(U/L)**	63	20–107
Aspartate aminotransferase **(U/L)**	56	6–44
Total Protein **(g/dL)**	7	6–8
Albumins **(g/dL)**	2.8	2.6–3.9
Urea (**mg/dL)**	22	25–70
Creatinine (**mg/dL)**	1.2	1–1.8
Calcium **(mg/dL)**	9.14	8–11
Phosphorus **(mg/dL)**	5.6	3–6.8

Computed tomography examination was performed under general infusion anaesthesia with intravenous propofol (2 mg/kg body weight (BW), after premedication with the use of dexmedetomidine (40 μg/kg BW, intramuscularly) and intubation. The examination was carried out with a 2-slice helical scanner (Siemens Somatom Emotion Duo, Siemens, Germany), with scanning parameters 130 kVp and 100 mAs, pitch 2,0 and reconstructed slice thickness 1.25 mm in the soft tissue window level (WL) 40 Hounsfield Unit (HU) window width (WW) 350 HU and bone tissue windows (WL 300 HU WW 1500 HU). The patient was placed in sternal recumbency with legs directed towards CT gantry. A scan of the abdominal cavity of the cat was performed prior to the administration of the contrast agent, within 30 s after its administration to visualize the cortical perfusion of the kidneys (nephrographic phase), and 5 min after its administration to visualize the secretory function of the kidney, together with the exact location of the renal pelvis and ureters. Iodine, non-ionic contrast agent (Ultravist 370, Bayer) at a dose of 700 mg iodine/kg BW was used, which corresponded to a dose of 2 ml/kg BW. The contrast was administered manually to the cephalic vein through a 20G intravenous cannula.

**Fig. 2:**
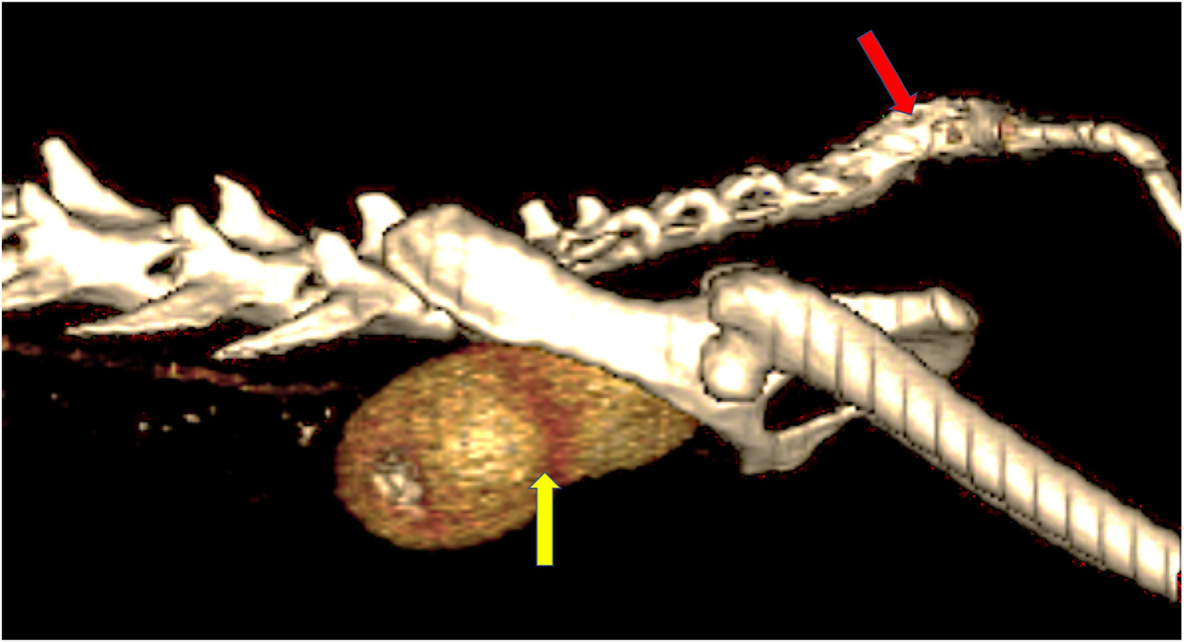
3D volume reconstruction (3D VR) of computed tomography scans. Sagittal plane, nephrographic phase (30 s after contrast agent administration), unusual kidney location (ectopia) visible, additionally: duplex kidney (yellow arrow). Deformation of the shape of the tail (red arrow)

**Fig. 3:**
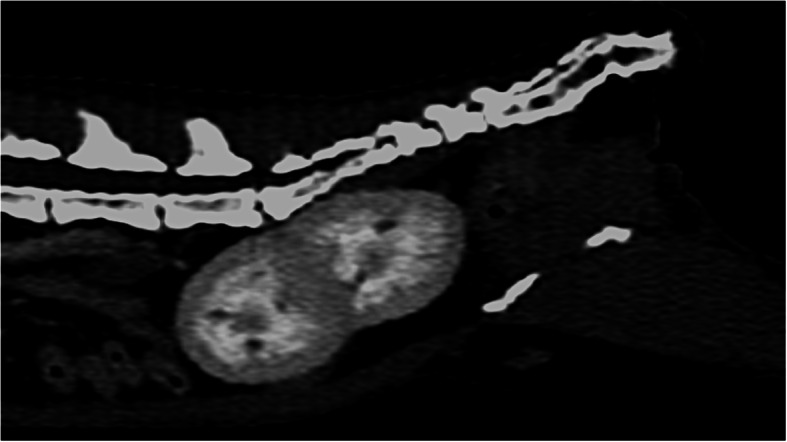
Multiplanar reconstructions of CT scans in the sagittal plane. Fusion of the kidneys with their ectopia. The nephrogram phase shows uniform renal enhancement prior to urine collecting in the renal pelvis

Computed tomography examination revealed the presence of an ectopic duplex kidney, whose cranial pole was adjacent to the bifurcation of the abdominal aorta, and the caudal pole was located in the pelvic cavity. The caudal part of the kidney corresponds to the left kidney, whose long axis and ureter both cross the midline migrating to the right, which corresponds to the renal ectopia. The presence of 2 independent, non-enlarged renal pelvises, without calcifications was confirmed. Two ureters, non-enlarged, shortened, crossed, but running correctly into the bladder were revealed on CT image. Contrast was simultaneously secreted by each kidney (Figs. 2 and 3).

**Fig. 4:**
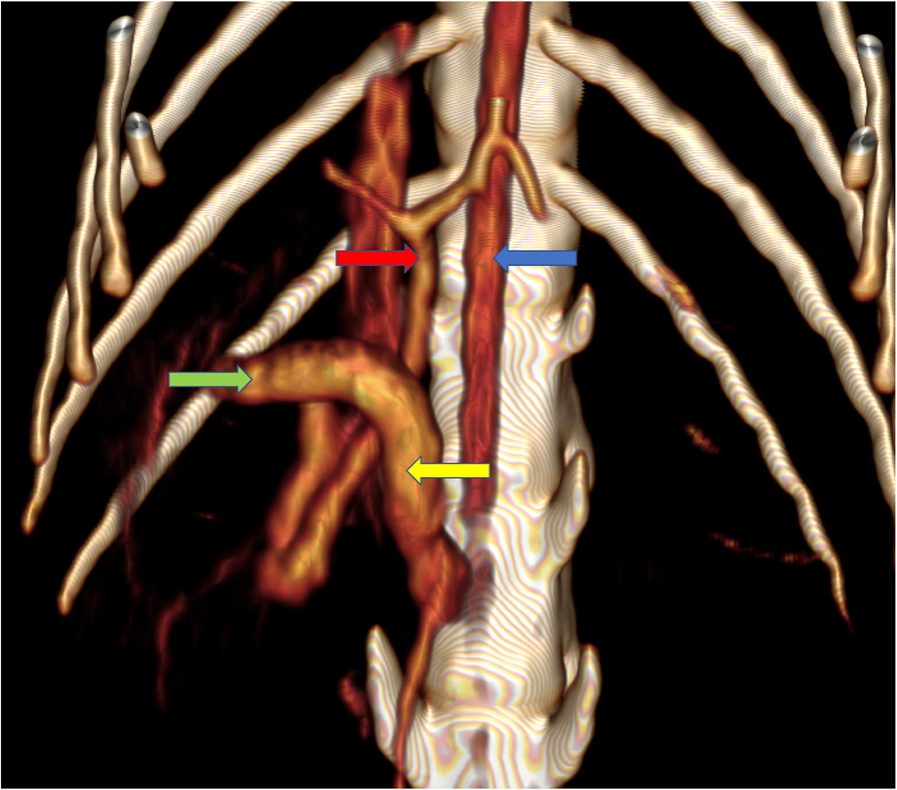
3D volume reconstruction (3D VR) of computed tomography scans. Dorsal plane, atypical portal vein visible without pathological shunts with systemic circulation vessels. Arrows: blue – aorta, yellow – portal vein, widened, green – portal vein, part before hepatic hilum, red – portal vein in hepatic hilum, dividing physiologically into two branches

**Fig. 5:**
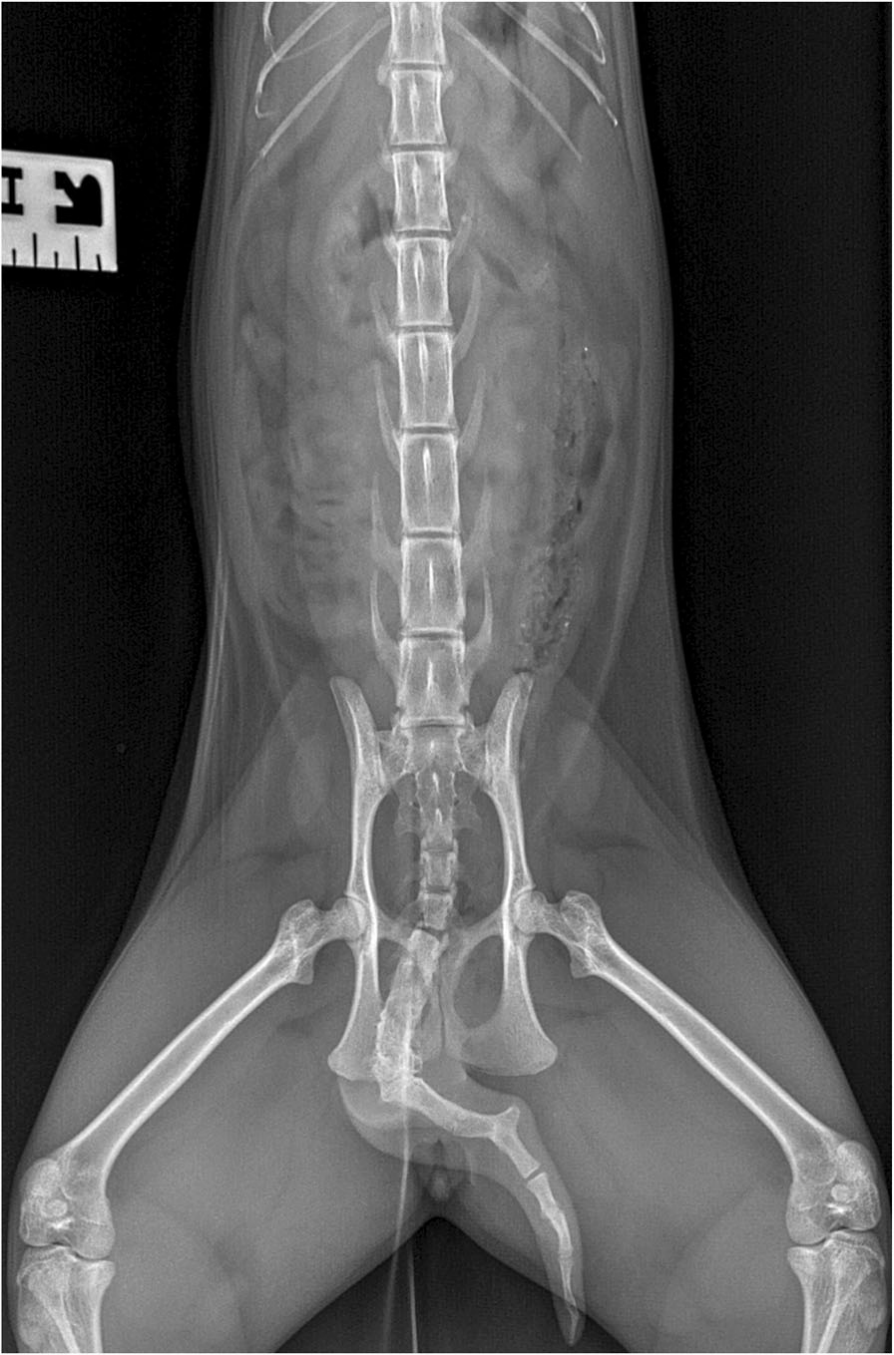
Ventrodorsal projection (VD) radiograph of the abdomen. A block of coccygeal vertebrae with shortening of the tail and twisting in the long axis

An unusual course and shape of the portal vein were also shown in CT images (Fig. 4). The portal vein, after reaching the spleen and pancreatic-duodenal veins, expanded to about 5.5 mm. At the level of the pylorus, the portal vein adjoined to dorsal wall of pylorus, and headed to the right, entered the liver parenchyma. In the area of the right liver lobe, the portal vein turned caudally, and then run again on the broadened trunk of the portal vein described above towards the liver gates. It entered the liver as a vessel with a diameter of about 3 mm and divided dichotomously. No abnormal vascular connections between the portal and systemic circulations were found. A block of tail vertebrae was also found, making the tail deformed and stiff, resulting in its serpentine shape (Fig. 5).

All CT and X-ray digital images were subjected to the analysis and post-processing using a dedicated imaging workstation and digital medicine viewer software (OsiriX MD 64-bit by Pixmeo, Switzerland).

## Discussion and conclusions

Physiologically, the left kidney of the cat is located caudally, close to the gastric fundus, caudally and medially to the head of the spleen, and laterally to the aorta. The right kidney cranial pole is found in the hepatic impression of the caudate liver lobe, dorsally and most often laterally to the duodenum, and laterally to the caudal vena cava. The normal kidney length ranges from about 3 cm to about 5.3 cm [13]. Their size depends on many factors, in neutered cats the kidneys are usually larger [14]. Larger kidneys are also more frequently found in males than in females [13].

An ectopic kidney occurs when the organ is in a non-physiological position. Crossed fused renal ectopia is a type of ectopia in which the kidney moves to the opposite side of the abdominal cavity, and the ureter crosses the midline of the body. The horseshoe kidney consists in the fusion of the renal poles through the isthmus made of renal parenchyma. There is also a simple ectopia in which the kidney is on the physiologically relevant side of the abdomen, but in a different topographic position [15]. In human medicine, the most commonly described type of CFRE is the shift of the left kidney, together with the ureter, to the right side of the abdomen and the fusion with the right kidney, which is in a physiological position. Unambiguous classification of some types of these anomalies is difficult due to the numerous classifications proposed in the literature. It is assumed that there are 6 types of CFRE in humans: Inferior crossed fusion; Sigmoid or S-shaped kidney; Lump kidney; Disc kidney; L-shaped or Tandem kidney; Superior ectopia type. The modified classification divides CFRE into: (1) crossed ectopia with fusion; (2) crossed ectopia without fusion; (3) bilateral crossed ectopia, and (4) solitary crossed ectopia [3]. Other human ectopic kidneys in the abdominal caudal region include pelvic kidney and crossed fused pelvic kidney (pancake kidney) [16].

In crossed ectopia with fusion, the migration of the left kidney to the right side is most often described in humans [3]. In the cases of CFRE reported to date in cats, the kidney fusion consisted in moving one of them to the contralateral one, located physiologically [3, 9, 10]. In the present case, both kidneys were ectopic, in addition a fusion and changes in the sides of the renal pelvis were observed. Moreover, the ureters were significantly shortened. Due to the multiplicity of types of ectopia described in humans and the occurrence of common features of several types of ectopia in the case being described, unambiguous naming of the type of ectopia was more difficult. According to the authors’ knowledge, renal ectopia co-occuring with portal vein and spinal abnormalities in the same patient has not been described in cats yet.

The CT image allows us to classify the diagnosed defect as S-shaped CFRE, but the fact that the ectopic kidney was in the pelvic cavity also allows it to be classified as a pancake kidney [3, 16]. Contrast enhanced CT examination allows seeing the kidneys and ureters in various planes allowing much more detailed diagnosis than ultrasound and excretory urography. This is the first description of such a case in the literature. More than 50% of patients with CFRE are asymptomatic, but about 50% of symptomatic patients have complications such as nephrolithiasis, hydronephrosis, urinary tract infections, or pyelonephritis [3]. By monitoring the clinical status of CFRE patients (i.e. capillary refill time, skin elasticity, sunken eyes, blood pressure, creatinine and urea levels, abdominal USG examination) the risk of these complications can be reduced. Measuring of the above mentioned parameters should be conducted periodically and any abnormalities should be corrected as soon as possible to prevent disease development. In the case described, the results of blood and urine tests as well as excretory urography did not indicate any disturbance of kidneys’ secretory function. The patient did not present clinical signs of renal dysfunction. In this case the detection of described abnormality was incidental, during a standard abdominal USG examination. It is possible that the lack of clinical symptoms and kidney dysfunction results from the shortening of the ureters, so that they run straight and do not wind up as in the case of some types of CFRE. Imaging of the above mentioned lesion may be helpful before abdominal surgery, as CFRE may be associated with various vascular and anatomical abnormalities (i.e. different vessel patterns, abnormal ureter course), which were confirmed in this case.

Apart from the confirmation of the defect in the renal structure, the CT examination allowed for an incidental diagnosis referring to the unusual course of the portal vein, without the presence of portal anastomoses and with a normal pattern of portal and venous liver vascularization. The most frequently reported defects of the portal vein are congenital and acquired portal shunts [17]. Rare cases of congenital absence of the portal vein (CAPV) have also been reported [17]. The anomaly diagnosed in the present patient has not been reported previously. Moreover, in this case, the third defect, in the form of a tail vertebral block and the deformation of the shape of the tail was also observed. Among described earlier anomalies of caudal vertebrae in cats, there were lack of the tail or reduction of the tail’s length with deformations in Manx breed [18, 19]. The most common abnormality is transitional vertebrae with the highest number of abnormalities at the sacrocaudal junction [19]. All reported defects did not affect the general condition of cats and were not associated with any clinical symptoms.

Crossed fused renal ectopia is a rare congenital anomaly and is easily detectable in an abdominal USG examination and CT, which allows more complete assessment of both anatomical relations and secretory function of the kidney. The occurrence of CFRE, abnormal portal vein and spinal malformation in a clinically healthy patient is the evidence that congenital malformations may simultaneously involve various structures and systems not directly related to each other, such as in the case presented. In these cases, blood and urine tests are of marginal significance, thus the diagnostic imaging is the most important examination in the accurate evaluation of the morphological and functional changes, especially contrast enhanced CT. Cats’ renal fusions should be considered in the differential diagnosis of caudal abdominal masses.

## Data Availability

The data used to support the findings of this study are available from the corresponding author upon request.

## Abbreviations

BCS: Body condition score
bpm: Beats per minute
CAPV: Congenital absence of the portal vein
CFRE: Crossed fused renal ectopia
CRT: Capillary refill time
CT: Computed tomography
FIV: Feline immunodeficiency virus
FeLV: Feline leukemia virus
Res: Resolution
RI: Resistance index
USG: Ultrasonographic
X – ray: Radiograph
